# Identification of Mild Freezing Shock Response Pathways in Barley Based on Transcriptome Profiling

**DOI:** 10.3389/fpls.2016.00106

**Published:** 2016-02-08

**Authors:** Xiaolei Wang, Dezhi Wu, Qian Yang, Jianbin Zeng, Gulei Jin, Zhong-Hua Chen, Guoping Zhang, Fei Dai

**Affiliations:** Zhejiang Key Lab of Crop Germplasm, Department of Agronomy, Zhejiang UniversityHangzhou, China

**Keywords:** barley (*Hordeum vulgare* L.), differentially expressed genes, freezing shock, RNA-sequencing, signaling pathway

## Abstract

Low temperature is a major abiotic stress affecting crop growth and productivity. A better understanding of low temperature tolerance mechanisms is imperative for developing the crop cultivars with improved tolerance. We herein performed an Illumina RNA-sequencing experiment using two barley genotypes differing in freezing tolerance (Nure, tolerant and Tremois, sensitive), to determine the transcriptome profiling and genotypic difference under mild freezing shock treatment after a very short acclimation for gene induction. A total of 6474 differentially expressed genes, almost evenly distributed on the seven chromosomes, were identified. The key DEGs could be classified into six signaling pathways, i.e., Ca^2+^ signaling, PtdOH signaling, CBFs pathway, ABA pathway, jasmonate pathway, and amylohydrolysis pathway. Expression values of DEGs in multiple signaling pathways were analyzed and a hypothetical model of mild freezing shock tolerance mechanism was proposed. Expression and sequence profile of *HvCBFs* cluster within *Frost resistance-H2*, a major quantitative trait locus on 5H being closely related to low temperature tolerance in barley, were further illustrated, considering the crucial role of *HvCBFs* on freezing tolerance. It may be concluded that multiple signaling pathways are activated in concert when barley is exposed to mild freezing shock. The pathway network we presented may provide a platform for further exploring the functions of genes involved in low temperature tolerance in barley.

## Introduction

Low temperature is one of the major adverse environmental factors that limit crop production. To cope with low temperature, plants have evolved various signal perception and response pathways, which subsequently lead to complicated biochemical and physiological changes, such as reprogramming of gene expression, modification of metabolic pathways, alteration in lipid composition, accumulation of osmoprotectants, and synthesis of antioxidants (Hughes and Dunn, 1996; Dai et al., 2009; Chinnusamy et al., 2010).

Low temperature perception is the first step for plants to develop the tolerance reactions, where a physical response is converted into complex biochemical and physiological processes (Ruelland and Zachowski, 2010). Temperature decrease can be sensed by plant cells through their membrane rigidification effect. Fatty acid desaturase (*FAD*) encode the enzymes that control the unsaturation of phospholipids and affect membrane fluidity (Miquel et al., 1993). In *Arabidopsis*, a *fad2* mutant defective in oleate desaturase exhibits plasma membrane rigidification and low temperature sensitivity (Miquel et al., 1993; Vaultier et al., 2006). Membrane rigidification-activated mechano-sensitive or ligand-activated Ca^2+^ channels lead to Ca^2+^ influx into cytosol. This was thought to be an important initial event to temperature change in plant cells, as calcium chelators and calcium channel blockers could prevent cold-induced protein phosphorylation and gene expression (Monroy et al., 1997; Chinnusamy et al., 2006; Winfield et al., 2010). The spatial and temporal patterns of Ca^2+^ signals in plant cells are characteristic for particular stimuli and can be interpreted by Ca^2+^-binding proteins (CBPs) that act as Ca^2+^ sensors including calmodulins (CaMs), calmodulin-like proteins (CMLs), calcineurin B-like proteins (CBLs), and calcium-dependent protein kinases (CDPKs; McCormack et al., 2005; Kaplan et al., 2006). After binding Ca^2+^, CBPs undergo a conformational change that enables them to activate or inactivate target proteins (DeFalco et al., 2010). For instance, CaMs bind to calmodulin-binding transcription factor (CAMTA) and CBLs interact with CBL-interacting protein kinases (CIPKs; Doherty et al., 2009; Batistic et al., 2010; Thoday-Kennedy et al., 2015). Afterwards, these downstream effectors initiate a series of events, resulting in extensive reprogramming of gene expression in response to low temperature (Winfield et al., 2010). Ca^2+^ signal can also be amplified through phospholipids such as phosphatidic acids (PtdOH), which are lipid secondary messengers mediating low temperature signaling in plants (Vergnolle et al., 2005; Chinnusamy et al., 2006). During low temperature treatment, PtdOH could be synthesized by two pathways: directly by the action of a PLD or by the combined action of a PLC, which produces diacylglycerol (DAG), followed by the action of a diacylglycerol kinase (DGK; Ruelland et al., 2002). Low temperature induced activation of both phospholipase pathways mainly depends on Ca^2+^ entry into cells and PLC activity accounts for almost 80% of low temperature-induced PtdOH (Ruelland et al., 2002).

The C-repeat binding factors (CBFs)-dependent pathway, which plays a central role among multiple low temperature regulatory pathways has been extensively studied and well-characterized (Chinnusamy et al., 2007). As members of a large AP2/EREBP family of DNA-binding proteins, CBFs are rapidly induced by low temperature and can bind to the DRE/CRT *cis*-element in the promoter regions of many cold responsive (COR) genes, which encode cryoprotective proteins for protecting cells against low temperature induced damage (Chinnusamy et al., 2003; Campoli et al., 2009). A common feature of *CBFs* in grass genomes is that *CBFs* are organized in clusters of tandemly duplicated paralogs (Tondelli et al., 2011). In barley, a quantitative trait locus on 5H, *Frost resistance-H2* (*Fr-H2*), contains a cluster of *HvCBFs* and largely contributes to low temperature tolerance (Francia et al., 2004; Knox et al., 2010). A recent study has identified the full-length sequences of *HvCBFs* in *Fr-H2* using a high-resolution genetic map of *Fr-H2* (Pasquariello et al., 2014).

Abscisic acid (ABA) accumulates when plants are exposed to low temperature, and is subsequently perceived by ABA receptors such as the proteins in the pyrabactin resistance/pyrabactin resistance 1-like/regulatory components of ABA receptor (PYR/PYL/RCAR) family. PYR/PYL/RCAR proteins can inhibit the activity of the clade A protein phosphatase type 2C (PP2C), relieving the repression of sucrose non-fermenting 1-related protein kinase 2 (SnRK2) activity by a clade A PP2C, which in turn phosphorylates abscisic acid responsive element (ABRE) binding protein/abscisic acid responsive element binding factor (AREB/ABF) to regulate the expression of low temperature responsive genes (Furihata et al., 2006; Ma et al., 2009; Park et al., 2009). A few of *COR* genes with ABRE in their promoters, such as *COR47*, can response to ABA and gain an increased expression level (Xiong et al., 2001). Moreover, ABA biosynthesis contributes to the maximum induction of low temperature responsive genes during the later stage of low temperature response (Zhang et al., 2014).

Blocking endogenous jasmonate biosynthesis and signaling renders plants hypersensitive to freezing stress, suggesting that jasmonate positively regulate freezing tolerance in plants (Hu et al., 2013). Jasmonate can be perceived by its receptor coronatine insensitive 1 (COI1) and subsequently facilitates the degradation of jasmonate-zim-domain proteins (JAZ), which act as repressors of jasmonate signaling via their physical interactions with a wide array of transcription factors (Thines et al., 2007; Yan et al., 2009).

Carbohydrate metabolism pathway was also altered in response to low temperature (Jiang et al., 2014). Starch degradation generates soluble sugars (such as sucrose, glucose, and trehalose), which can modulate cellular osmotic potential to protect plasma membrane from low temperature damage (Bogdanovic et al., 2008).

To better understanding the functional characterization of low temperature induced genes in cereals, more attention should be paid to choice of genotypes and experimental conditions, such as treatment and sampling time (Campoli et al., 2009). It has been reported that many *COR* genes show the maximal expression within 2 days after low temperature treatment (Ganeshan et al., 2008), therefore, sampling after short-term treatment could be ideal to capture the transcriptome profile of low temperature perception and signaling. Our previous study was focused on the change of chlorophyll fluorescence parameters during recovery after freezing shock in barley (Dai et al., 2007), however, no research has been done on the systematic transcriptome profiling of freezing shock response genes in barley. RNA-sequencing (RNA-Seq) is an efficient tool for gene expression profiling studies as well as simultaneous identification of mutations (Morozova et al., 2009), and it has been applied in low temperature tolerance research of many plant species, such as *Solanum lycopersicoides* (Chen et al., 2015), *Chrysanthemum nankingense* (Ren et al., 2014), and *Camellia sinensis* (Wang et al., 2013).

Therefore, we herein performed mild freezing shock treatment and RNA-Seq analysis using two barley cultivars, Nure (low temperature tolerant) and Tremois (low temperature sensitive; Francia et al., 2004; Rizza et al., 2011) to explore the perception and signaling pathways activated by short-term freezing treatment, in order to draw an outline of early regulatory gene network in response to freezing shock in barley.

## Materials and methods

### Plant materials and mild freezing shock treatment

Two barley (*Hordeum vulgare* L.) cultivars, Nure, and Tremois, were used in this study. Nure is a winter barley cultivar originated from Italy with superior low temperature tolerance, while Tremois is a spring barley cultivar originated from France with inferior low temperature tolerance (Francia et al., 2004). Compared with Tremois, Nure has higher winter survival rate, better frost tolerance, and more accumulation of COR proteins (Francia et al., 2004; Rizza et al., 2011).

Seeds of both cultivars, kindly provided by Dr. Luigi Cattivelli in CRA Agricultural Research Council, Italy, were sown in plastic pots (170 mm × 220 mm) filled with peat in November 24th 2012. Plants were grown in a glasshouse at Zhejiang University, Hangzhou, China. Temperature was set at 20/14°C (day/night), and relative humidity was 75%. Mild freezing shock treatment was conducted on three-leaf-stage seedlings under natural condition. As the freezing shock treatment, plants were moved to the open air in a fine day of winter from 5 p.m. to 8 a.m. of the next day, with the temperature fluctuation from -2 to 0°C. The plants kept in glasshouse were used as control. The plants of both control and treatment were sampled immediately after freezing shock treatment (at 8 a.m.) for analysis.

### Sampling and RNA isolation

The third fully expended leaves of 10 seedlings were collected, mixed and flash frozen in liquid nitrogen. Total RNA was extracted from an approximately 0.5 g frozen sample with TRIzol Reagent (Invitrogen, Carlsbad, CA), purified with RNeasy Mini Kit (Qiagen, Germantown, MD) and quality-checked with the Agilent 2100 Bioanalyser (Agilent Technologies, Palo Alto, CA), and the RNA samples were frozen at −80°C until used.

### Library construction, sequencing, and data processing

Library construction and sequencing were conducted as previously described in Dai et al. (2014). Briefly, the poly-A containing mRNA was enriched from total RNA by poly-T oligo-attached magnetic beads, and then fragmented into small pieces randomly. Using the fragments as templates, first-strand cDNA was synthesized with random hexamer-primers. Second-strand cDNA was synthesized using DNA polymerase I, dNTPs, and RNase H. The products were amplified by PCR and purified after end-repairing and adaptor ligations with the Illumina TruSeq™ RNA Sample Preparation Kit (Illumina, San Diego, CA). PCR products were loaded onto Illumina HiSeq2000 platform (Illumina, San Diego, CA) for 2 × 100 bp paired-ends sequencing. Raw reads were trimmed by removing all adaptor sequences, empty reads and low quality reads (Q < 30 and length < 50 bp) to obtain clean reads. The clean reads were mapped to the assembly genome of *cv*. Morex (Mayer et al., 2012) downloaded from Ensembl Genomes 2013 (http://plants.ensembl.org/index.html, Kersey et al., 2014) using Tophat v2.0.8b with default parameters (Trapnell et al., 2009). Cuffdiff of Cufflinks v2.1.1 was used to assemble the mapped reads, estimate the abundances, and analyze differentially expressed genes (DEGs) (Roberts et al., 2011).

Duplicated reads in the BAM files created by TopHat were removed by MarkDuplicates program of Picard v1.7 (http://picard.sourceforge.net). Uniquely mapped single-end and paired-end results were used for the SNVs (single nucleotide variants) and *indel* (insertion or deletion variant) calling. Raw SNVs and *indel* were called using SAM-tools mpileup and bcftools (Li and Durbin, 2009), and then were filtered with mapping quality score ≥ 50 and reads coverage > 4.

### Identification of the DEGs and quantitative RT-PCR analysis

Transcripts abundance of each gene was normalized by using the fragments per kilobase per million map reads (FPKM) method (Li et al., 2014). The ratio of [FPKM (treatment)]/[FPKM (control)] or [FPKM (control)]/[FPKM (treatment)] was calculated as fold-change and the subtraction of |[FPKM (treatment)]—[FPKM (control)]|as number-change. According to previous reports, both fold-change and number-change should be taken into account (Zenoni et al., 2010; Li et al., 2014). In our study, DEGs were determined by the following criterion: “fold-change > 2 and number-change > 20” or “fold-change > 4 and number-change > 2.” This threshold took both highly expressed genes with relatively smaller fold change and lowly expressed genes with relatively greater fold change into consideration. An online software jvenn (http://bioinfo.genotoul.fr/jvenn/example.html) was used to generate Venn diagram (Bardou et al., 2014).

Expression of 10 randomly selected DEGs was determined by quantitative real-time PCR (qRT-PCR) to validate RNA-Seq data. All the primers are listed in Table S1. qRT-PCR was performed on a CFX96 system (Bio-Rad, USA) as described by Zeng et al. (2014) with some modifications. The *actin* gene was used as an internal control (Zheng et al., 2011).

### Gene annotation, GO enrichment, and KEGG analysis

Protein sequences of barley and *Arabidopsis* were downloaded from Ensembl Genomes 2013 (http://plants.ensembl.org/index.html), and Blastp of Blast v2.2.28 was performed with the parameters “-max_target_seqs 1 -evalue 1E-5” to annotate barley homologous genes to *Arabidopsis* (Altschul et al., 1990). Setting “max target seqs” as “1,” only the best match result was considered.

Using nucleotide sequences of DEGs as queries, Blastx of Blast2GO v3.0 was performed with default parameters to align against NCBI non-redundant (nr) protein database for homology search. GO annotation and KEGG pathways mapping of DEGs were performed using Blast2GO v3.0 (Conesa et al., 2005).

### Presentation of potential mild freezing shock signaling pathways in Figure 1

Ca^2+^ signaling pathway was presented based on the reports of Batistic et al. (2010), Doherty et al. (2009), Hunt et al. (2004), Lecourieux et al. (2006), McCormack et al. (2005), and Townley and Knight (2002). PtdOH signaling was drawn as described by Arisz et al. (2013) with some modifications. *CBFs*-dependent low temperature response pathway was drawn referring to Chinnusamy et al. (2007) and Guan et al. (2013). ABA pathway was presented based on the reports of Acharya et al. (2013), Ding et al. (2015), Nambara and Marion-Poll (2005), Valdés et al. (2012), and Yoshida et al. (2015). Jasmonate pathway and amylohydrolysis pathway were presented mostly based on KEGG map00592 and map00500, respectively.

**Figure 1:**
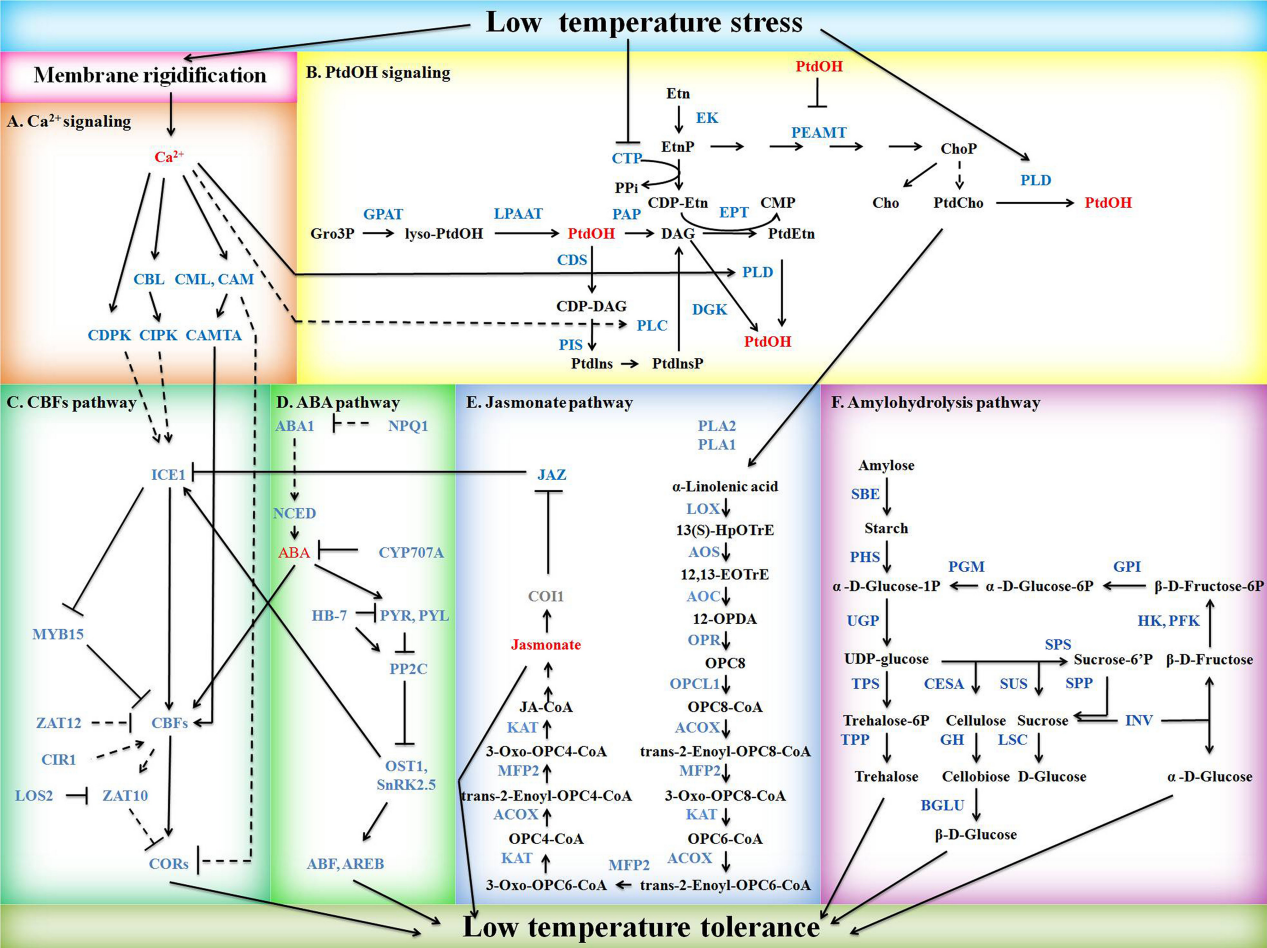
**Schematic diagram of potential mild freezing shock signaling pathways in barley**. Signaling molecules, DEGs, non-DEGs, and metabolites or others are shown in red, blue, gray, and black, respectively. Solid arrows indicate positive regulation whereas lines ending with a bar stand for negative regulation. Regulations are direct (solid line) or indirect (dash line). **(A)** Ca^2+^ signaling. CAM, calmodulin; CAMTA, calmodulin-binding transcription factor; CBL, calcineurin B-like protein; CDPK, calcium-dependent protein kinase. CIPK, CBL-interacting protein kinase; CML, calmodulin-like protein. **(B)** PtdOH signaling. CDS, cytidine diphosphate-diacylglycerol synthase; CTP, phosphorylcholine cytidylyltransferase; DGK, diacylglycerol kinase; EK, ethanolamine kinase; EPT, cytidine diphosphate-ethanolamine phosphotransferase; GPAT, glycerol 3-phosphate acyltransferase; LPAAT, lysophosphatidic acid acyltransferase; PAP, phosphatidic acid phosphatase; PEAMT, phosphoethanolaminemethyltranferase; PIS, phosphatidylinositol synthase; PLC, phospholipase C; PLD, phospholipase D; PtdOH, phosphatidic acid. **(C)** CBFs pathway. CBF, C-repeat binding factor; CIR1, circadian 1; COR, cold responsive genes; ICE1, inducer of CBF expression 1; LOS2, low expression of osmotically responsive genes 2; MYB15, Myb domain protein 15. **(D)** ABA pathway. ABA1, ABA deficient 1; ABF, ABA-responsive elements-binding factor; AREB, ABA-responsive element-binding protein; CYP707A, cytochrome P450-family 707-subfamily A; HB-7, homeobox-7; NCED, nine-cis-epoxycarotenoid dioxygenase; NPQ1, non-photochemical quenching 1; OST1, open stomata 1; PP2C, protein phosphatase 2C; PYL, pyrabactin resistance 1-like; PYR, pyrabactin resistance; SnRK2.5, sucrose non-fermenting 1-related protein kinase 2.5. **(E)** Jasmonate pathway. ACOX, acyl-CoA oxidase; AOC, allene-oxide cyclase; AOS, allene oxide synthase; COI1, coronatine insensitive 1; JAZ, jasmonate-zim-domain protein 11; KAT, ketoacyl-CoA acyltransferase; LOX, linoleate 13S-lipoxygenase; MFP2, enoyl-CoA hydratase; OPCL1, OPC-8:0 CoA ligase 1; OPR, 12-oxophytodienoate reductase; PLA1, phospholipase A1; PLA2, phospholipase A2. **(F)** Amylohydrolysis pathway. BGLU, beta-glucosidase; CESA, cellulose synthase; GH, 4-beta-D-glucan 4-glucanohydrolase; GPI, glucose-6-phosphate isomerase; HK, hexokinase; INV, invertase; LSC, levansucrase; PFK, phosphofructokinase; PGM, phosphoglucomutase. PHS, alpha-glucan phosphorylase; SBE, starch branching enzyme; SPP, sucrose-phosphate phosphatase; SPS, sucrose-phosphate synthase; SUS, sucrose synthase; TPP, trehalose-6-phosphate phosphatase; TPS, trehalose-6-phosphate synthase; UGP, UDP-glucose pyrophosphorylase.

### Identification of DEGs encoding *HvCBFs* within *Fr-H2* locus

Using nucleotide sequences of *HvCBFs* within *Fr-H2* reported by Pasquariello et al. (2014) as a query, Blastn of Blast v2.2.28 was conducted with the parameters “-max_target_seqs 1 -evalue 1E-5” in order to identify the *HvCBFs* cluster in our DEGs (Altschul et al., 1990). Distributions of *HvCBFs* in *FR-H2* locus shown in **Figure 4** refer to Pasquariello et al. (2014).

### Genetic difference analysis

In order to understand the genetic difference between Nure and Tremois, |log_2_[FPKM(Nure)/FPKM(Tremois)]|was calculated for both treatment and control. Here, the greater is the value, the greater is genotypic difference. The number of DEGs on a certain value of |log_2_[FPKM(Nure)/FPKM(Tremois)]|was counted to form two trendlines in Figure S1, which revealed changes of DEGs number as genetic difference become greater. Only |log_2_[FPKM(Nure)/FPKM(Tremois)]|not larger than 4 were presented.

## Results

### RNA-Seq performance

After a very short acclimation for gene induction, over 113 million raw reads (10.52 Gb) for the four libraries of two barley genotypes in control and mild freezing shock treatment were yielded by the 100-bp paired-end transcriptome sequencing (Table S2). The sequences of raw data have been deposited in the National Center for Biotechnology Information Sequence Read Archive, www.ncbi.nlm.nih.gov (accession nos. SAMN03952824 and SAMN03952823). After removing adaptor sequences, empty reads, and poor quality reads, about 84.1% raw reads were determined as clean reads (Table S2). Approximately 88.0, 86.6, 86.6, and 85.8% of clean reads for Nure in control (Nure_C), Nure in treatment (Nure_T), Tremois in control (Tremois_C), and Tremois in treatment (Tremois_T) were mapped to the assembly genome of *cv*. Morex, and yielded 40,321, 37,714, 38,997, 39,065 transcripts, respectively (Table S2).

### Overall analysis of DEGs

**Figure 2:**
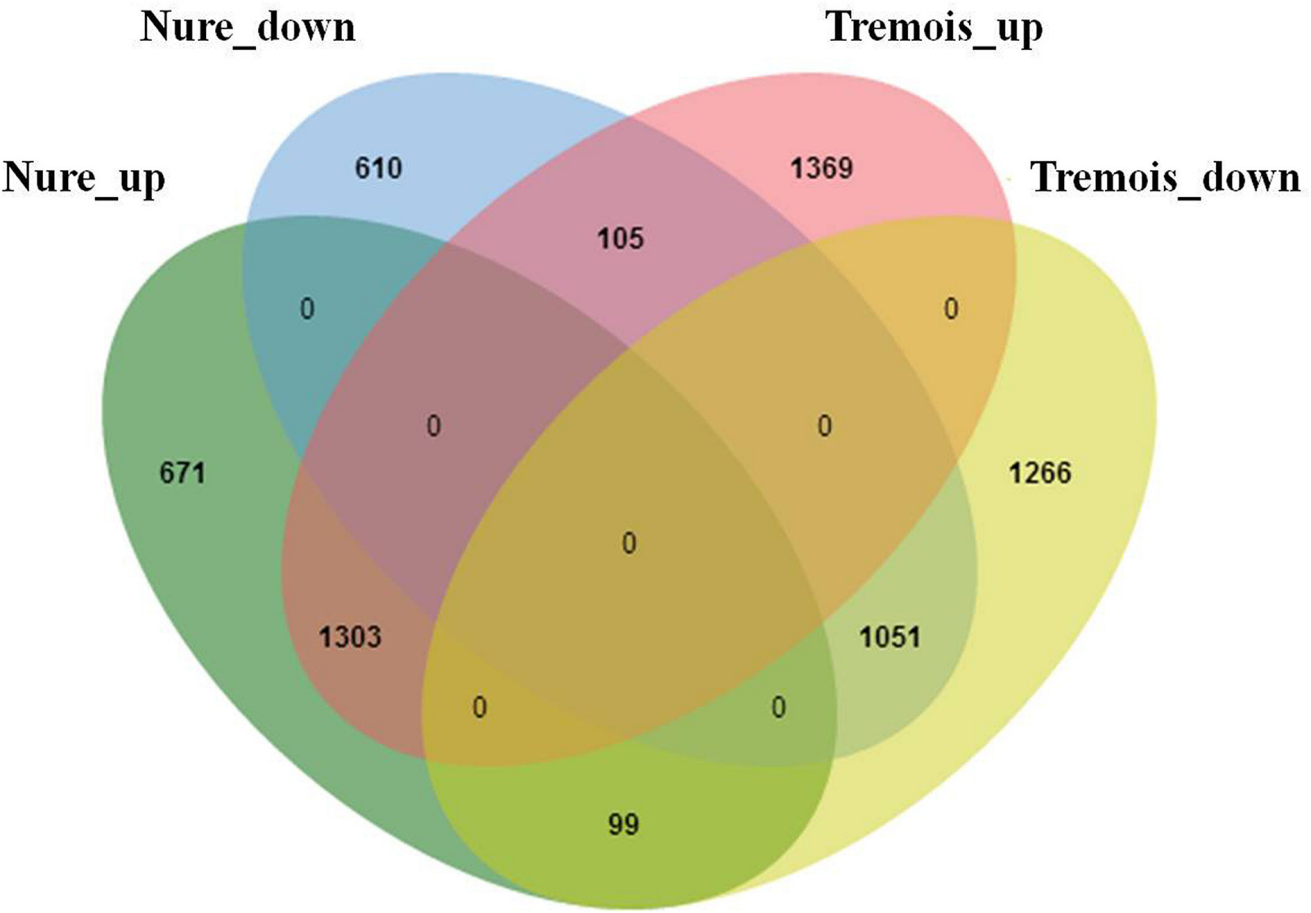
**Overlapping of differentially expressed genes (DEGs) in Nure and Tremois after freezing shock**. Nure_up, DEGs up-regulated in Nure. Nure_down, DEGs down-regulated in Nure. Tremois_up, DEGs up-regulated in Tremois. Tremois_down, DEGs down-regulated in Tremois.

Based on the criterion for screening DEGs, a total of 6474 genes were determined to be differentially expressed under low temperature treatment in comparison to the control (Table S3). Overall, these DEGs were almost evenly distributed on the seven chromosomes in barley (Table 1). Nure had less DEGs than Tremois, and both genotypes had a relatively higher proportion of up-regulated DEGs (Figure 2). There were 1303 up-regulated DEGs and 1051 down-regulated DEGs in both genotypes (Figure 2). However, 770 and 1474 DEGs were up-regulated only in Nure and Tremois, respectively (Figure 2). As many as 1266 DEGs were unchanged in Nure but down-regulated in Tremois (Figure 2). There were 3906 DEGs and 4465 DEGs in control and treatment showing genotypic difference between Nure and Tremois (Figure S1).

**Table 1:** **Chromosome distribution of differentially expressed genes (DEGs) in low temperature signaling pathways**.

Chromosome^*^	**1H**	**2H**	**3H**	**4H**	**5H**	**6H**	**7H**	**Unanchored**	**Total**
Ca^2+^ signaling	10	5	6	7	11	2	7	0	48
PtdOH signaling	6	3	5	1	2	5	1	0	23
CBFs pathway	0	2	1	1	9	4	3	0	20
ABA pathway	1	7	3	2	6	3	1	0	23
Jasmonate pathway	1	4	2	7	6	4	7	0	31
Amylohydrolysis pathway	7	13	12	7	12	8	8	1	68
Total DEGs	816	1041	961	773	1019	750	909	205	6474

*DEGs, differentially expressed genes. Unanchored, DEGs not anchored to any of seven chromosomes*.

* *The length of chromosome referred to the synthetic assembly of cv. Morex (Ensembl Genomes 2013, http://plants.ensembl.org/info/website/ftp/index.html)*.

**Figure 3:**
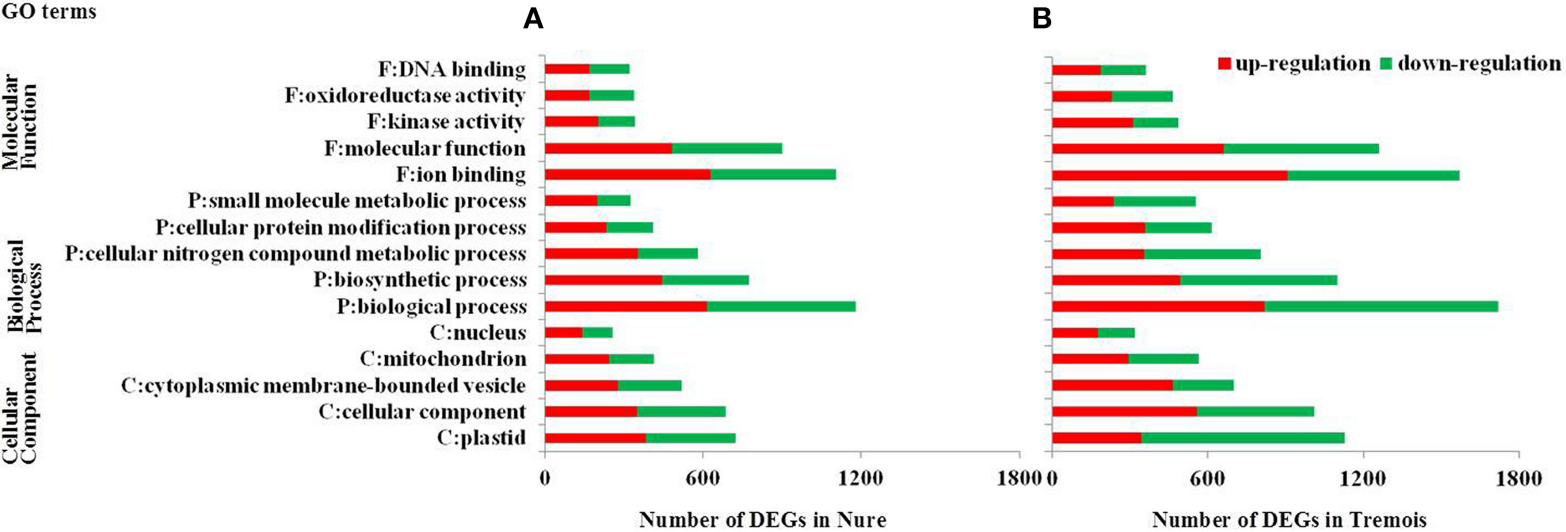
**Gene Ontology (GO) analysis of differentially expressed genes (DEGs) in Nure (A) and Tremois (B)**. F, Molecular Function; P, Biological Process; C, Cellular Component; DEGs, differentially expressed genes. Only the top five GO terms in each of the three major categories, molecular function, biological process, and cellular component, are presented.

GO (gene ontology) analysis of the whole 6474 DEGs was performed and the top five GO terms in each of the three major categories, molecular function, biological process and cellular component, were presented in Figure 2. In both genotypes, “ion binding,” “biological process,” and “plastid” were the most enriched terms in molecular function, biological process and cellular component, respectively, followed by “molecular function,” “biosynthetic process,” and “cellular component,” and then “kinase activity,” “cellular nitrogen compound metabolic process,” and “cytoplasmic membrane-bounded vesicle” (Figure 3). Then KEGG (Kyoto encyclopedia of genes and genomes) pathway enrichment was further investigated. A total of 1058 DEGs encoding 464 enzymes were assigned to 129 KEGG pathways, including lipid, carbohydrate, amino acid, energy, and other metabolic process (Table S4). DEGs encoding transferases made up the largest proportion, followed by hydrolases and oxidoreductases (Figure S2).

DEGs involved in low temperature perception, Ca^2+^ signaling, PtdOH signaling, CBFs pathway, ABA pathway, jasmonate pathway, and amylohydrolysis pathway were identified according to gene annotations and resulted in a hypothetical model of mild freezing shock tolerance mechanism in barley (Figure 1). The Ca^2+^, PtdOH, CBFs, and ABA pathways were mainly based on DEGs homologous to *Arabidopsis* identified by Blastp and relevant studies in *Arabidopsis*, while jasmonate and amylohydrolysis pathway were mainly based on KEGG maps. Annotations and FPKMs of DEGs in Figure 1 are listed in Table S5.

In order to assess the reliability of RNA-Seq data, we randomly selected 10 DEGs to investigate their expression in Nure and Tremois using qRT-PCR. There were highly significant correlations in both Nure and Tremois between RNA-Seq data and qRT-PCR results (Figure S3), indicating the high reliability of RNA-Seq data in the current study.

### DEGs involved in Ca^2+^ signaling

A total of 48 DEGs involved in Ca^2+^ signaling were identified, i.e., 5 *CaMs*, 23 *CMLs*, 2 *CBLs*, 2 *CDPKs*, 2 *CAMTAs*, and 14 *CIPKs* (Figure 1A, Table S5). One *CAM*, one *CDPK*, three *CIPKs* and ten *CMLs* were up-regulated, while one *CAM*, one *CML*, and six *CIPKs* were down-regulated in both genotypes (Table S5). Two *CAMs*, two *CMLs*, two *CBLs*, and two *CIPKs* decreased in Nure but were unchanged in Tremois, while one *CAM*, one *CIPK*, one *CDPK*, two *CAMTAs*, and seven *CMLs* were unchanged in Nure but increased in Tremois after mild freezing shock (Table S5). *CML4* (MLOC_71936) and *CML6* (MLOC_62796) were up-regulated in Nure but unchanged in Tremois, while *CML22* (MLOC_61579), *CIPK26* (MLOC_63556), and *CIPK11* (MLOC_12021) were unchanged in Nure but down-regulated in Tremois (Table S5). There was difference in the distribution of the 48 DEGs: 11 DEGs were anchored to chromosome 5H but only 2 DEGs were found in chromosome 6H (Table 1).

### DEGs involved in PtdOH signaling

We identified 23 DEGs involved in PtdOH signaling (Figure 1B, Table S5). One *PLD*, one glycerol 3-phosphate acyltransferase (*GPAT*), one *DGK*, and two phosphatidic acid phosphatases (*PAPs*) increased, while one *GPAT*, one ethanolamine kinase (*EK*), one phosphorylcholine cytidylyltransferase (*CTP*), one cytidine diphosphate diacylglycerol synthase (*CDS*), and two phosphoethanolaminemethyltranferases (*PEAMTs*) decreased in both Nure and Tremois after mild freezing shock (Table S5). One *PLD* and one *GPAT* were down-regulated in Nure but unchanged in Tremois, while one *PLD*, one lysophosphatidic acid acyltransferase (*LPAAT*), one *PAP*, one *EK*, one phosphatidylinositol synthase (*PIS*), and one *PLC* were unchanged in Nure but up-regulated in Tremois (Table S5). The DEG encoding lipid phosphate phosphatase (*LPP3*, MLOC_72673) in *Arabidopsis* was down-regulated in Nure but up-regulated in Tremois (Table S5). *LPAAT* (MLOC_69398), cytidine diphosphate-ethanolamine phosphotransferase (*EPT*, MLOC_19179), and *PLC* (MLOC_58308) were unchanged in Nure but showed a 55.5, 66.1, and 51.0% decrease in Tremois after mild freezing shock (Table S5). As for the chromosome distribution, 1H harbors the most DEGs of PtdOH signaling pathway (Table 1).

### DEGs involved in *CBFs* pathway

The 20 DEGs involved in *CBFs* pathway were interspersed on all seven chromosomes except 1H, where 5H having 9 DEGs (Table 1). Inducer of CBF expression 1 (*ICE1*), *CBFs*, circadian 1 (*CIR1*), low expression of osmotically responsive genes 2 (*LOS2*), and *CORs* were found to be positive regulators, while Myb domain protein 15 (*MYB15*), *ZAT12*, and *ZAT10* were negative regulators of *CBFs*-dependent low temperature response pathway (Figure 1C).

DEG (MLOC_46407) homologous to *ICE1* in *Arabidopsis* was identified to be unchanged in Nure but up-regulated in Tremois (Table S5). Four *HvCBFs*, e.g., *HvCBF3* (MLOC_4956), *HvCBF6* (MLOC_63682), *HvCBF9* (MLOC_41994), and *HvCBF14* (MLOC_59224) were up-regulated in both genotypes, while *HvCBF1* (MLOC_75886) and *HvCBF4B* (MLOC_54227) were unchanged and down-regulated in Nure respectively, but up-regulated in Tremois. (Table S5). Two homologous genes of *CIR1* in *Arabidopsis* were found out in our DEGs (MLOC_57684 and MLOC_62877) but their expression levels showed a dramatic difference. MLOC_62877 increased in both genotypes while MLOC_57684 was unchanged in Nure and decreased in Tremois by 53.6% after mild freezing shock (Table S5). MLOC_37167, a homologous DEG of *LOS2* in *Arabidopsis* exhibited an 87.5% decrease in Nure after exposure to low temperature (Table S5). Two CORs (MLOC_14884 and MLOC_70331) were both up-regulated by low temperature with different expression patterns. MLOC_14884, had higher expression level than MLOC_70331 in all samples (Table S5). Both MLOC_14884 and MLOC_70331 showed lower expression values under control and higher expression values under treatment in Nure (Table S5).

Three DEGs (MLOC_34646, MLOC_43806 and MLOC_73037) homologous to *MYB15* in *Arabidopsis* were collectively up-regulated in Nure but unchanged in Tremois (Table S5). Three *ZAT10* homologs (MLOC_54674, MLOC_65033 and MLOC_70662) were up-regulated in all samples with an increase of at least 6.32 (Table S5). Three DEGs (MLOC_19822, MLOC_63032, and MLOC_76196) homologous to *ZAT12* in *Arabidopsis* showed low expression levels under normal condition and were up-regulated by mild freezing shock in both cultivars (Table S5).

**Figure 4:**
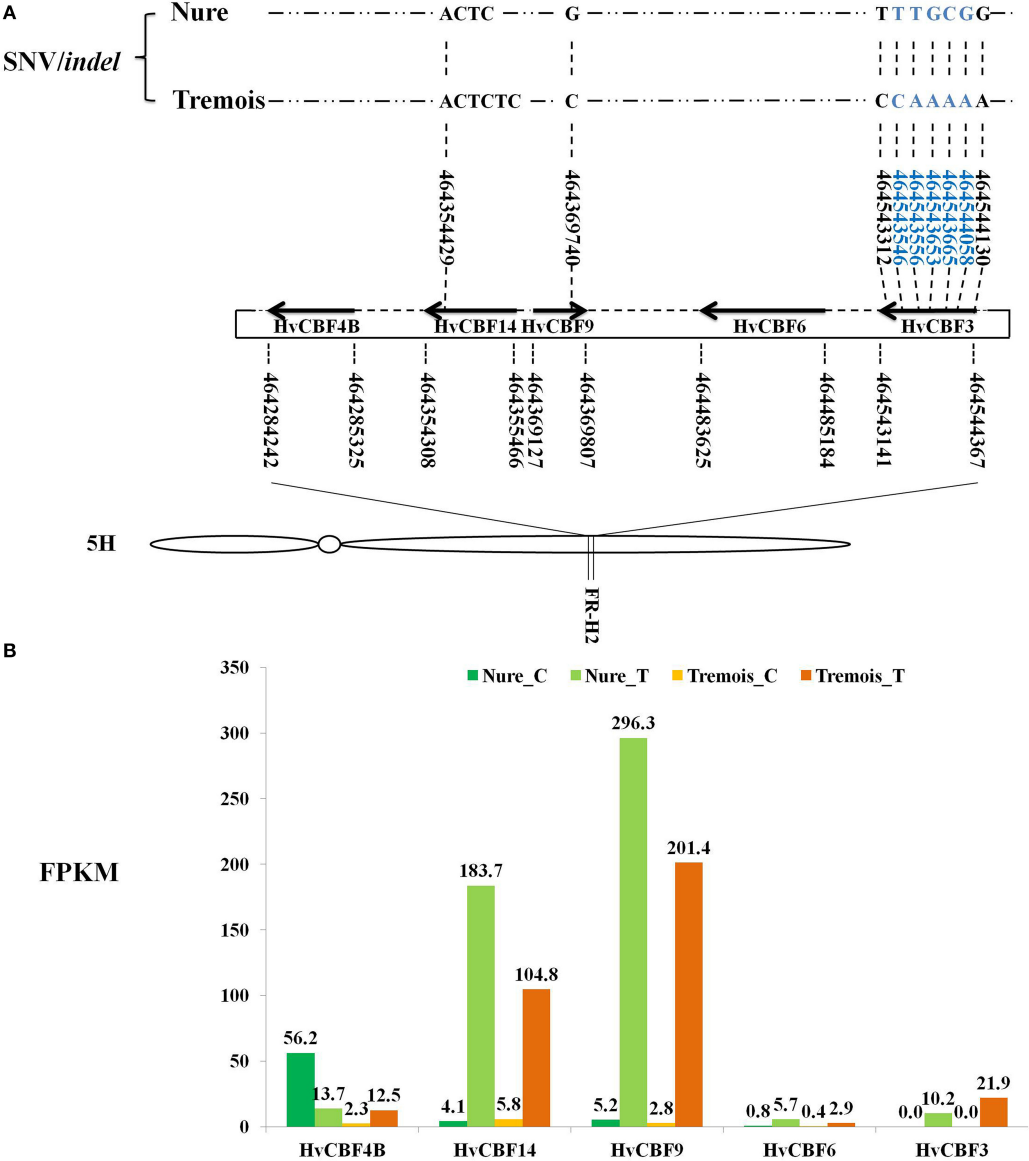
**Feature of *HvCBFs* in *FR-H2* locus of Chromosome 5H in barley. (A)** Distribution and SNV/*indel* characteristics of *HvCBFs* in *FR-H2* locus. Boundaries of *HvCBFs* in *FR-H2* locus are illustrated by bold arrows. The numbers under and above gene names are physical locations of genes and SNV/*indel*, respectively. Right and left arrows show positive and negative strand of *HvCBFs* on the assembly genome. SNVs/*indels* sequence differed between Nure and Tremois are shown, and SNVs/*indels* in blue locate in the coding domain sequence (CDS) regionof *HvCBF3*. *Indel*, insertion or deletion variant. SNV, single nucleotide variant. Distributions of *HvCBFs* in *FR-H2* locus refer to Pasquariello et al. (2014). **(B)** Expression values of *HvCBFs* in *FR-H2* locus. Fragments per kilo base per million map reads (FPKMs) of samples are labeled above each bar. Nure_C, Nure in control. Nure_T, Nure in treatment. Tremois_C, Tremois in control. Tremois_T, Tremois in treatment.

Among the DEGs, Five *HvCBFs* were identified within the *Fr-H2* locus by Blastn. *HvCBF9* is on the positive strand while *HvCBF4B, HvCBF14, HvCBF6*, and *HvCBF3* are on the negative strand (Figure 4A). A startling up-regulation of *HvCBF9* were indicated as its FPKMs were 5.2 and 2.8 under control while 296.3 and 201.4 after mild freezing shock to Nure and Tremois, respectively (Figure 4B). *HvCBF14* also increased largely after mild freezing shock, with a 43.8- and 17.1-fold increase in Nure and Tremois, respectively (Figure 4B, Table S5). An SNV of *HvCBF9* was identified as guanine (G) in Nure and cytosine (C) in Tremois at physical location 464369740 on 5H (Figure 4A). An insertion of “TC” within *HvCBF14* in the physical location of 46435429 in Tremois compared with Nure was discovered (Figure 4A). A total of seven SNVs within *HvCBF3* were identified, five of which were located in the coding domain sequence (CDS) area (Figure 4A). Only SNVs/*indels* causing sequence differences between Nure and Tremois were shown in Figure 4.

### DEGs involved in ABA pathway

Abscisic acid deficient 1 (*ABA1*), 9-cis-epoxycarotenoid dioxygenase (*NCED*), *PYR, PYL*, open stomata 1 (*OST1*), sucrose non-fermenting 1-related protein kinase 2.5 (*SnRK2.5*), *ABF*, and *AREB* are positive regulators while non-photochemical quenching 1 (*NPQ1*), cytochrome P450-family 707-subfamily A (*CYP707A*), homeobox-7 (*HB-7*), and protein phosphatase 2C (*PP2C*) are negative regulators in ABA biosynthesis and signaling (Figure 1D). Among positive regulators, *PYL5* (MLOC_71349) and *OST1* (MLOC_69212) were up-regulated in Nure but unchanged in Tremois, while *ABF3* (MLOC_20326 and MLOC_66546) were unchanged in Nure but down-regulated in Tremois (Table S5). Among negative regulators, *HB-7* (MLOC_69710) was down-regulated in Nure but unchanged in Tremois and *HAI3* (MLOC_8131), which encoded a cluster A PP2C, being unchanged in Nure but up-regulated in Tremois (Table S5). Furthermore, according to the results of Blastn, the up-regulated *HvCBF3* shared the similar nucleotide sequences with *CBF4* in *Arabidopsis*, which positively regulated ABA signaling (Figure 1D). The 23 DEGs in ABA pathway were largely anchored to chromosomes 2H and 5H of assembly genome of Morex (Table 1).

### DEGs involved in jasmonate pathway

In total, 23 DEGs encoding 10 key enzymes during endogenous jasmonate biosynthesis from PtdCho and 8 DEGs encoding 4 JAZ proteins (negative regulators in jasmonate signaling) were identified (Figure 1E, Table S5). Among enzymes participating in jasmonate biosynthesis, phospholipase A (*PLA2*, MLOC_6428 and MLOC_24486) were up-regulated in Nure but unchanged in Tremois, while linoleate 13S-lipoxygenase (*LOX*, MLOC_64972) was up-regulated in Nure and down-regulated in Tremois (Table S5). Approximately 80% of DEGs encoding enzymes involved in jasmonate biosynthesis were up-regulated (Table S5). A DEG (MLOC_59388) encoding homolog of *JAZ12* in *Arabidopsis* was unchanged in Nure but up-regulated in Tremois (Table S5).

### DEGs involved in amylohydrolysis pathway

A total of 68 DEGs encoding 17 enzymes involved in amylohydrolysis were identified. Starch branching enzyme (*SBE*, MLOC_63722), beta-glucosidase (*BGLU*, MLOC_79756), invertase (*INV*, MLOC_56803), phosphofructokinase (*PFK*, MLOC_15972), and glucose-6-phosphate isomerase (*GPI*, MLOC_1497) were up-regulated in Nure but unchanged in Tremois. However, cellulose synthases (*CESAs*, MLOC_7825, and MLOC_43749), *BGLUs* (MLOC_37976, MLOC_12571, and MLOC_37740), sucrose-phosphate synthase (*SPS*, MLOC_63746), sucrose-phosphate phosphatase (*SPP*, MLOC_6173), *INVs* (MLOC_60935, MLOC_72784 and MLOC_79590), and *GPIs* (MLOC_61974 and MLOC_40899) were unchanged in Nure but down-regulated in Tremois (Table S5). Regarding the chromosome distribution of DEGs of amylohydrolysis pathway, 2H had the largest number of DEGs, followed by 3H and 5H (Table 1).

## Discussion

In the current study, we performed a RNA-Seq analysis to reveal the transcriptome reprogramming of barley plants under mild freezing shock after a very short acclimation for gene induction, which is different from the normal crop environmental conditions. We used such a treatment to mimic the natural condition with gradually change of temperature but non-lethal to winter barley, i.e., Nure, in order to potentially trigger as many genes as possible in response to low temperature. Moreover, this is not a study on the function of specific genes, so we pooled samples from 10 plants, but not single leaf from one for RNA-seq, which could average the differences between the responses of individual seedlings to the mild freezing treatment. And also, we only randomly selected some DEGs and conducted a “technical” validation of the fold-change ratios of the sequenced transcripts. This type of approach is widely used in many other studies, e.g., Yokosho et al. (2014).

An early response genes network of mild freezing shock was investigated by classifying DEGs into different freezing shock response pathways, named Ca^2+^ signaling, PtdOH signaling, CBFs pathway, ABA pathway, jasmonate pathway, and amylohydrolysis pathway (Figure 1). Membrane rigidification may be the first reaction to low temperature perception in plants, and reduced membrane fluidity is coupled with enhanced low temperature inducibility due to the action of a number of genes (Miquel et al., 1993). Four DEGs encoding homologs of *FAD* in *Arabidopsis* nearly collectively showed higher FPKMs under treatment in freezing tolerant cultivar Nure than freezing sensitive cultivar Tremois (Table S3), suggesting that higher content of unsaturated fatty acid after freezing shock may contribute to the superior freezing tolerance in Nure. This was consistent with previous study that plants preferentially accumulate polyunsaturated fatty acids under low temperature and genetically increasing of unsaturated fatty acids could enhance cold tolerance, and chilling tolerant rice cultivars contained greater proportion of unsaturated fatty acids than sensitive ones (Kodama et al., 1994; Ariizumi et al., 2002).

As a ubiquitous intracellular second messenger, Ca^2+^ participates in plant freezing response not only via Ca^2+^ signature but also by the cross-talk with other signaling pathways. Ca^2+^ may modulate PtdOH signaling by activating PLC and PLD, two pivotal enzymes in PtdOH synthesis (Hunt et al., 2004; Lecourieux et al., 2006). CAM is a potential negative regulator of *COR*, as its overexpression represses low temperature-induced *COR* expression in plants (Townley and Knight, 2002). *CAM7* (MLOC_19924) and *CAM9* (MLOC_65903) were down-regulated in freezing tolerant accession Nure and *CAM7* (MLOC_36991) was up-regulated in freezing sensitive varieties Tremois, which consists with the higher COR expression in Nure, suggesting the potential negative regulation of CAM on freezing tolerance in barley (Table S5). The cross-talk between Ca^2+^ signaling and CBFs pathway is also evident by the positive regulation of CAMTA on freezing induced expression of *CBF1* and *CBF2* in *Arabidopsis* (Doherty et al., 2009). In the present study, *CMTA4* (MLOC_44450 and MLOC_58709) were unchanged in Nure but up-regulated in Tremois, which may explain the higher expression value of *HvCBF1* under treatment in Tremois.

PtdOH signaling is also involved in mild freezing shock response. Low temperature can inhibit the activity of CTP, resulting in a limited availability of cytidine diphosphate-ethanolamine (CDP-Etn) and an accumulation of DAG as precursor for PtdEtn synthesis, the latter of which could be an additional source for freezing induced PtdOH through DGK activity (Inatsugi et al., 2002; Arisz et al., 2013). Decreased *CTP* (MLOC_19066), *EPT* (MLOC_19179) and increased *DGK* (MLOC_58803) after freezing shock indicated the presence of a similar regulation mechanism in barley (Table S5). Besides, reduced CTP may cause the accumulation of EtnP, which can be repeatedly methylated by PEAMT to form ChoP and then PtdCho (Figure 1B). As the rate-limiting enzyme for PtdCho synthesis, PEAMT (MLOC_15214 and MLOC_66415) showed a 58.0 and 560% higher FPKM under treatment in Nure than in Tremois, respectively (Table S5). PtdCho can then be catalyzed by PLD to generate PtdOH (Figure 1B).

Chromosome 5H of barley is very important for cold-responsive CBFs pathway, as 9 out of the 20 DEGs involved in CBFs pathway were anchored to it (Table 1) and the *HvCBFs* are clustered within *Fr-H2* locus on 5H. Among the five identified *HvCBFs* located in *Fr-H2, HvCBF14* and *HvCBF9* may be the major transcription factors for freezing tolerance. It was evident that FPKMs are increased sharply after freezing shock and the higher transcript level in freezing tolerant variety Nure (Figure 4). The expression difference of *HvCBFs* between Nure and Tremois may be partly related to the variation of gene copy number. For example, multiple copies of *HvCBF4* were found in Nure as compared to a single copy in Tremois (Knox et al., 2010). Though an *indel* in *HvCBF14* and a SNV in *HvCBF9* led to sequence differences between Nure and Tremois, these sequence variations may not result in amino acid change, because both variable sites are located in the 3′ end of CDS rather than in the coding region (Figure 4). Furthermore, two subgroups of the five *HvCBFs* can be divided according to the phylogenetic analysis in previous reports (Skinner et al., 2005): *HvCBF6* and *HvCBF3* belong to *HvCBF3*-subgroup, while *HvCBF4B, HvCBF14* and *HvCBF9* are classified into *HvCBF4*-subgroup. We postulate that *HvCBF4*-subgroup possibly contributes more to freezing shock tolerance than *HvCBF3*- subgroup in barley based on their expression values (Figure 4). Moreover, it has been reported that *HvCBFs* in *Fr-H2* could be affected by the major vernalisation gene (*VRN-H1)* on 5H in barley (Stockinger et al., 2007; Comadran et al., 2012). However, in the current study, none of the three genes determining growth habit in the vernalization pathway, *HvVRN1, HvVRN2*, and *HvVRN3/HvFT1* (Comadran et al., 2012), belonged to the 6474 DEGs, which may due to the short-term freezing shock treatment, sampling tissues or other factors, such as photoperiod (Stockinger et al., 2007).

Although, ICE1 can bind to the promoter of *CBF3* and enhance its expression in *Arabidopsis*, it is only slightly up-regulated by low temperature. It was reasoned that the freezing induced posttranslational modification of ICE1 such as phosphorylation is essential for its activation on CBFs (Chinnusamy et al., 2003). Furthermore, a well-known protein kinase in ABA signaling, SnRK2.6/OST1, was reported to be activated by low temperature in an ABA-independent mechanism and can phosphorylate and stabilize ICE1 to positively modulate CBFs pathway in *Arabidopsis* (Ding et al., 2015). DEGs (MLOC_69212, MLOC_11726, and MLOC_3013) homologous to *OST1* in *Arabidopsis* were up-regulated by mild freezing shock and Nure always showed a greater FPKM (Table S5). It has been proposed that, low temperature may alter the conformation of a sensor protein, which could be one or more of the PYL proteins, to activate OST1 (Ding et al., 2015). It has been reported that PYL5 in *Arabidopsis* was able to modulate drought resistance by inhibiting clade A PP2Cs and the inhibition could be ABA-independent (Santiago et al., 2009; Hao et al., 2011). Coincidentally, DEGs (MLOC_71349 and MLOC_65591) homologous to *PYL5* in *Arabidopsis* were up-regulated after freezing shock with larger increase in Nure (Table S5). More speculatively, we infer that, PYL5 (MLOC_71349 and MLOC_65591) in barley may be multiple sensory proteins that involves in the OST1-mediated CBFs dependent pathway in response to low temperature. This hypothesis may also be partly supported by the previous report that ABA receptors are candidates to improve abiotic stress tolerance of crops and constitutive overexpression of a cytosolic ABA receptor (OsPYL/RCAR5) in rice confers drought and salt tolerance (Kim et al., 2014).

As the pivot point of mild freezing response, CBFs pathway interacts with other signaling pathways to form an overall freezing tolerance in plants. Apart from the cross talk with PtdOH pathway and ABA pathway, CBFs pathway is also modulated by jasmonate. Three genes (MLOC_18524, MLOC_4800, and MLOC_80547) homologous to jasmonate receptor *COI1* in *Arabidopsis* were detected to be uninfluenced by low temperature (data not shown as they were not DEGs). Among the 8 DEGs encoding homolog of JAZ in *Arabidopsis*, 7 DEGs showed higher FPKM in Nure under treatment (MLOC_61774, MLOC_10653, MLOC_45755, MLOC_11970, MLOC_59389, MLOC_9995, and MLOC_37630), 2 DEGs were up-regulated in Nure but unchanged in Tremois (MLOC_61774 and MLOC_10653) (Table S5). Given the inhibition of JAZ on ICE1 (Figures 3C,E; Table S5; Hu et al., 2013), our result may indicate that the expression profile of the DEG (MLOC_46407) homologous to ICE1 in *Arabidopsis*, which was unchanged in Nure but up-regulated in Tremois.

Soluble sugar, such as trehalose, act as osmoprotectant in freezing tolerance by lowering osmotic potential to protect membranes, enzymes, and other structures against irreversible damage and denaturation (Bartels and Sunkar, 2005). Previous study shows that overexpression of two crucial trehalose synthetases, trehalose 6-phosphate phosphatase (TPP) and trehalose 6-phosphate synthase (TPS), enhances freezing tolerance in plants (Pramanik and Imai, 2005; Miranda et al., 2007). We also found that *TPS* (MLOC_11773) and three out of the four *TPPs* (MLOC_11773, MLOC_49094, and MLOC_52798) in barley increased significantly after freezing shock (Table S5).

In conclusion, multifarious pathways are activated in concert to perceive and transduce the signal in barley exposed to mild freezing shock. Besides, cross-talk occurred among different signaling pathways in response to mild freezing shock. The pathway network we obtained may provide a platform for further exploring the functions of genes involved in low temperature tolerance in barley.

## Author contributions

FD conceived and designed this project. XW, GJ, and FD carried out the bioinformatics analysis. FD, XW, DW, QY, and JZ performed all the experimental parts. GZ and ZHC participated in its design and coordination. XW, FD, DW, ZC, and GZ wrote the manuscript. All authors read and approved the final manuscript.

### Conflict of interest statement

The authors declare that the research was conducted in the absence of any commercial or financial relationships that could be construed as a potential conflict of interest.

## Supplementary material

Figure S1 **Genotype difference analysis of differentially expressed genes (DEGs) in Nure and Tremois**. FPKM: fragments per kilobase per million map reads. FPKM = 10^9^ × total_ fragments_in_exons/total_fragments_counted/transcript_length.

Figure S2 **Enzyme code distribution of differentially expressed genes (DEGs) in Nure and Tremois**.

Figure S3 **Correlation of gene expression ratio between RNA-Seq and qRT-PCR in Nure (A) and Tremois (B)**.

Table S1 **Primers of differentially expressed genes (DEGs) selected for quantitative real-time PCR (qRT-PCR) expression analysis**.

Table S2 **Mapping of RNA-Seq data to barley cv. Morex WGS contigs**.

Table S3 **Annotations and expression values of the 6474 differentially expressed genes (DEGs)**.

Table S4 **KEGG overview of differentially expressed genes (DEGs)**.

Table S5 **Expression values of differentially expressed genes (DEGs) in low temperature signaling pathways**.

## Abbreviations

ABA1: abscisic acid deficient 1
ABRE: abscisic acid responsive element
AREB/ABF: abscisic acid responsive element binding protein/abscisic acid responsive element binding factor
CaM: calmodulin
CAMTA: calmodulin-binding transcription factor
CBFs: C-repeat binding factors
CBLs: calcineurin B-like proteins
CBPs: Ca^2+^-binding proteins
CDP-Etn: cytidine diphosphate-ethanolamine
CDPKs: calcium-dependent protein kinases
CDS: coding domain sequence
CIPKs: calcineurin B-like protein-interacting protein kinases
CIR1: circadian 1
CMLs: calmodulin-like proteins
COI1: coronatine insensitive 1
COR: cold responsive genes
CYP707A: cytochrome P450-family 707-subfamily A
DAG: diacylglycerol
DEGs: differentially expressed genes
DGK: diacylglycerol kinase
FAD: fatty acid desaturase
FPKM: fragments per kilobase per million map reads
*Fr-H2*: *Frost resistance-H2*
GO: gene ontology
HB-7: homeobox-7
ICE1: inducer of CBF expression 1
indel: insertion or deletion variant
JAZ: jasmonate-zim-domain protein
KEGG: Kyoto encyclopedia of genes and genomes
LOS2: low expression of osmotically responsive genes 2
MYB15: Myb domain protein 15
NCED: 9-cis-epoxycarotenoid dioxygenase
NPQ1: non-photochemical quenching 1
nr: non-redundant
Nure_C: Nure in control
Nure_T: Nure in treatment
OST1: open stomata 1
PLC: phospholipase C
PLD: phospholipase D
PP2C: protein phosphatase type 2C
PPIs: polyphosphoinositides
PtdCho: phosphatidylcholine
PtdEtn: phosphatidylethanolamine
PtdOH: phosphatidic acid
PYL: pyrabactin resistance PYR-like
PYR: pyrabactin resistance
RCAR: regulatory components of ABA receptor
RNA-Seq: RNA sequencing
SnRK2: sucrose non-fermenting 1-related protein kinase 2
SNVs: single nucleotide variants
TPP: trehalose 6-phosphate phosphatase
TPS: trehalose 6-phosphate synthase
Tremois_C: Tremois in control
Tremois_T: Tremois in treatment.
