# Supplementary figures and images for: Identification of Mild Freezing Shock Response Pathways in Barley Based on Transcriptome Profiling
Source: Front Plant Sci. 2016 Feb 8;7:106. doi: 10.3389/fpls.2016.00106 (PMC4744895; doi:10.3389/fpls.2016.00106)

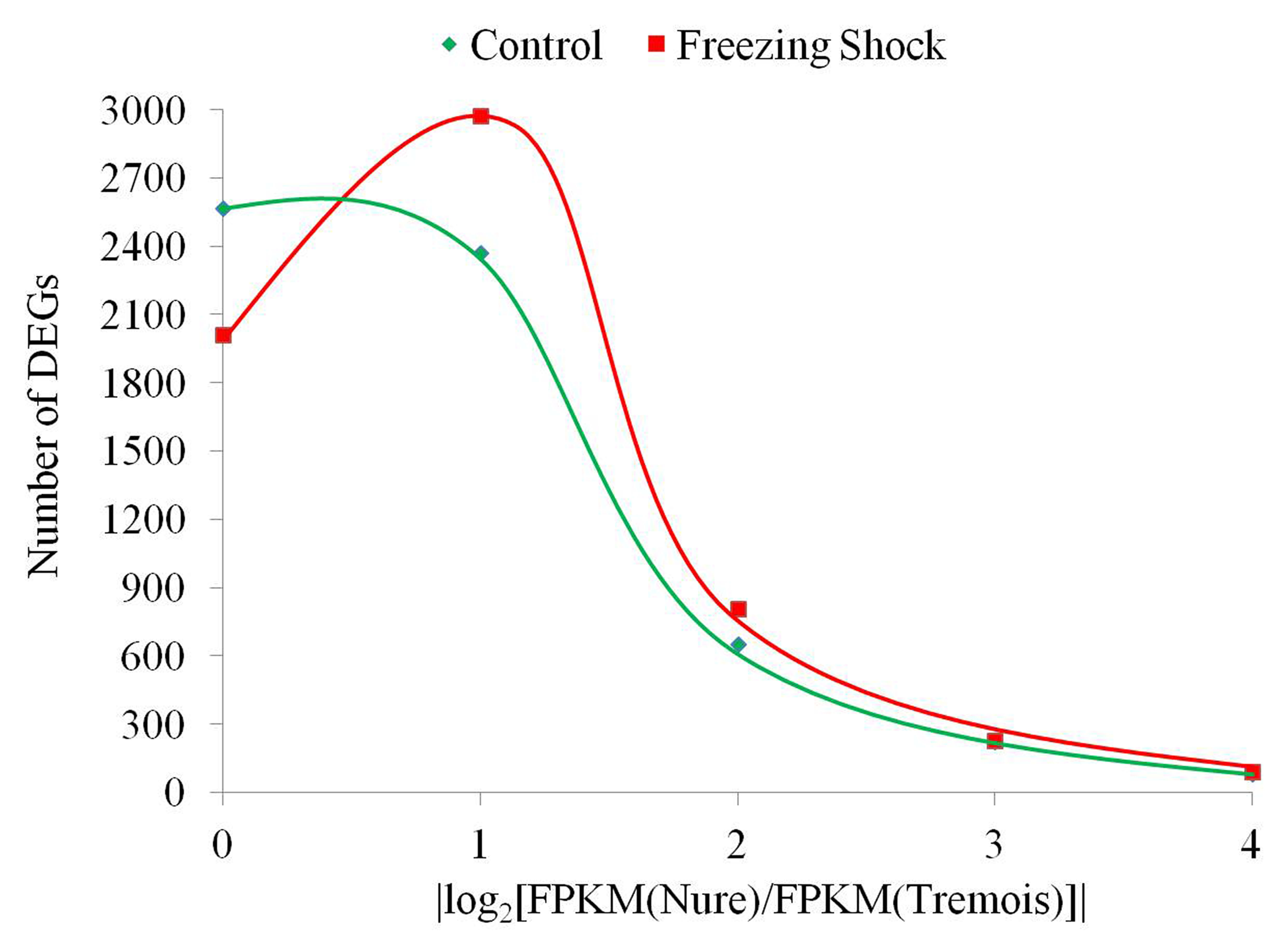

Supplement: Figure S1 — Genotype difference analysis of differentially expressed genes (DEGs) in Nure and Tremois. FPKM: fragments per kilobase per million map reads. FPKM = 109 × total_ fragments_in_exons/total_fragments_counted/transcript_length. [file Image1.JPEG]

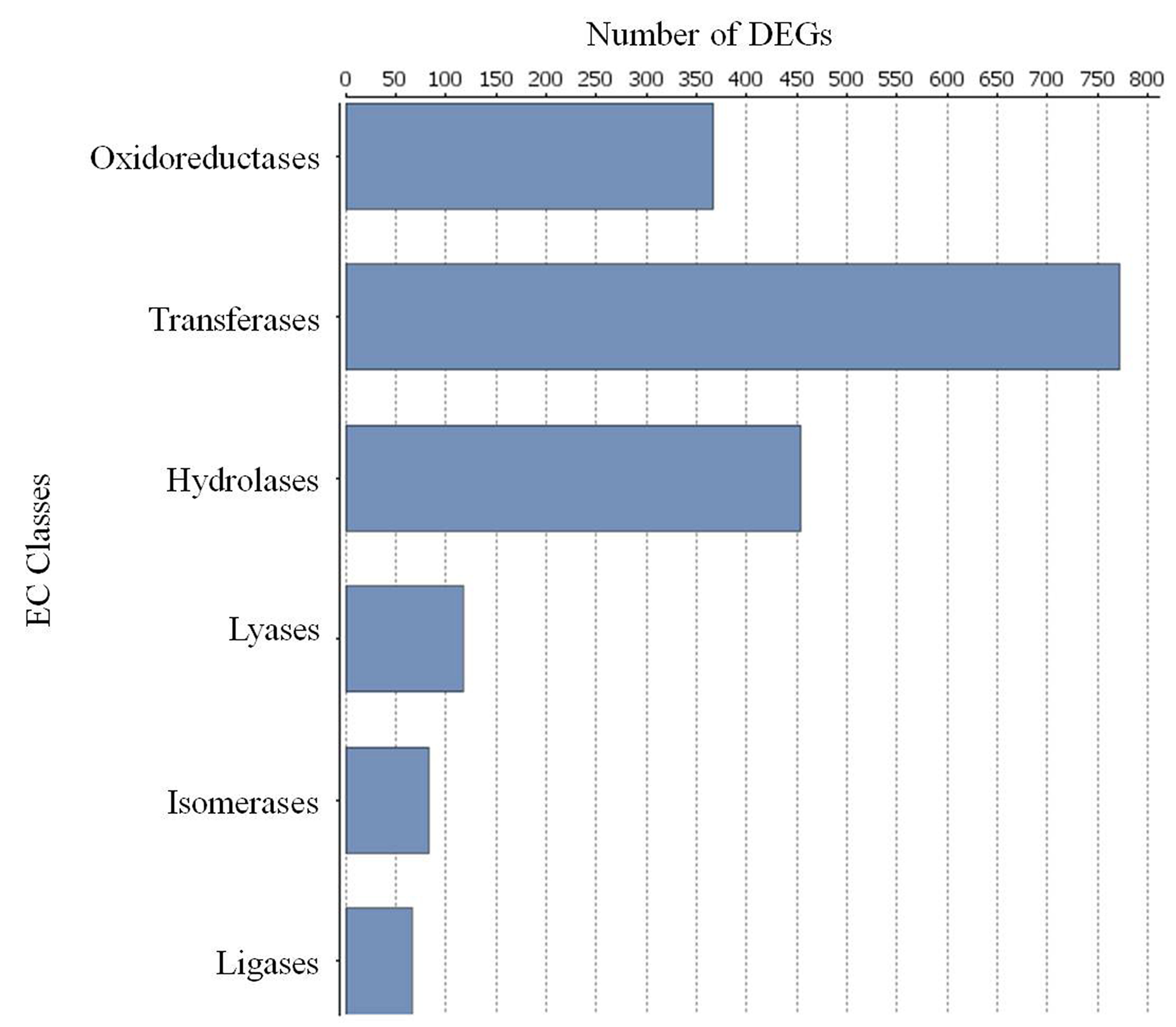

Supplement: Figure S2 — Enzyme code distribution of differentially expressed genes (DEGs) in Nure and Tremois. [file Image2.JPEG]

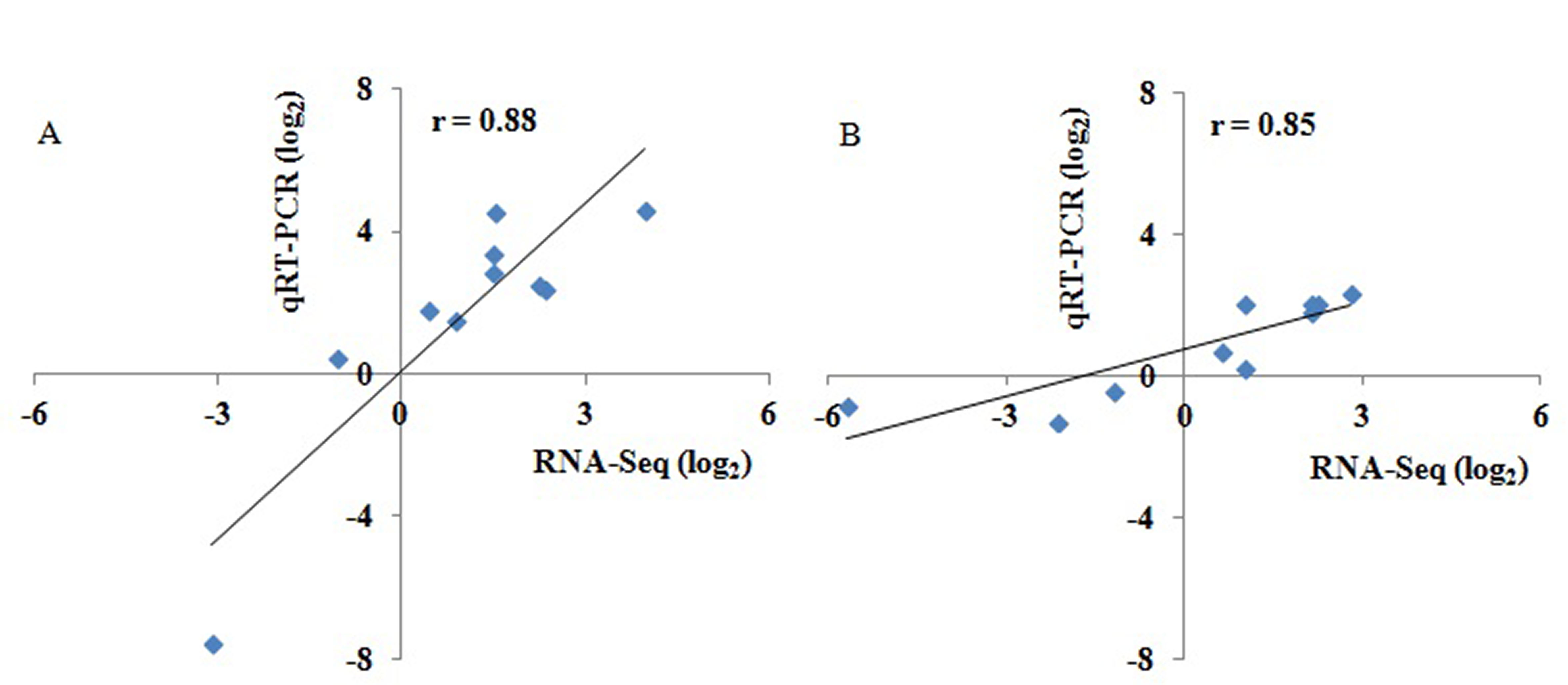

Supplement: Figure S3 — Correlation of gene expression ratio between RNA-Seq and qRT-PCR in Nure (A) and Tremois (B). [file Image3.jpg]
