# Supplementary material for: Identification of Mild Freezing Shock Response Pathways in Barley Based on Transcriptome Profiling
Source: Front Plant Sci. 2016 Feb 8;7:106. doi: 10.3389/fpls.2016.00106 (PMC4744895; doi:10.3389/fpls.2016.00106)
Supplement: Table S1 — Primers of differentially expressed genes (DEGs) selected for quantitative real-time PCR (qRT-PCR) expression analysis. [file Table1.DOCX]

**Table S1. Primers of differentially expressed genes (DEGs) selected for quantitative real-time PCR (qRT-PCR) expression analysis.**

| Barley_gene_ID | Barley_gene_name | Gene_by_annotation | Forward primer sequence (5'-3') | Reverse primer sequence (5'-3') |
| --- | --- | --- | --- | --- |
| MLOC _66415 |  | PEAMT | GCAGAAGAGCTACTGGGAGG | TGATGACGCTGTCGATGAAGT |
| MLOC_14884 | DHN8 | COR47 | GGAGCACCCAGTCATACCAG | GATCGGTGCAGCTTGGAGAA |
| MLOC_64943 |  | ABA1 | ACAAGGAACCTGCTGGTGG | GTGACACGACCTTTTCCCCA |
| MLOC_64972 | LOX2.1 | LOX | ATGCTGACGGCGACCAAG | GTCACCGTGGCCTTCATCTC |
| MLOC_36991 | CAM | CAM7 | CCATCGACTTCCCAGAGTTCC | GGATCATCTCGTCCACCTCC |
| MLOC_56293 | PLD | PLDα1 | GAACCTCCACGTCACCATCT | ACGCCCAACACGAGTTTTCT |
| MLOC_69212 |  | OST1 | GATGGAGCGGTACGAGGTG | TCCAGAACCGCAGATCCTTTC |
| MLOC_58709 |  | CMTA4 | TCCTCTCTCAATGCAGGTCATC | GACGGTGCGAAGTCTGTGAG |
| MLOC_14726 |  | NCED6 | AGAAGAAGCCCACCCATCAC | CAAAATTGCTCTCGGTGGGG |
| MLOC_63523 |  | PFK | GGTGGACATCCTGAAGCAGA | GGTCGTAGGAGCAGAGGATG |
|  | Actin^*^ |  | GACTCTGGTCATGGTGTCAGC | GGCTGGAAGAGGACCTCAGG |

^*^ The actin gene was used as an internal control.
