# Supplementary material for: Identification of Mild Freezing Shock Response Pathways in Barley Based on Transcriptome Profiling
Source: Front Plant Sci. 2016 Feb 8;7:106. doi: 10.3389/fpls.2016.00106 (PMC4744895; doi:10.3389/fpls.2016.00106)
Supplement: Table S2 — Mapping of RNA-Seq data to barley cv. Morex WGS contigs. [file Table2.DOCX]

**Table S2. Mapping of RNA-Seq data to barley cv. Morex WGS contigs.**

|  | Raw data and mapping statistics | | | | | |  | Mapped gene, transcript, and SNV/*indel* statistics | | |  | Data accession |
| --- | --- | --- | --- | --- | --- | --- | --- | --- | --- | --- | --- | --- |
| Sample | Raw reads | Raw bases (Gb) | Clean reads | Clean bases (%) | Mapped  reads (%) | Reads mapped in proper pairs |  | Mapped  genes | Mapped transcripts | Number of SNVs/*indels* |  |  |
| Nure_C | 31,912,536 | 2.97 | 27,698,840 | 83.65 | 88.03 | 21,600,448 |  | 18,938 | 40,321 | 36,647 |  | SAMN03952824 |
| Nure_T | 24,818,882 | 2.31 | 19,787,144 | 75.06 | 86.56 | 14,826,532 |  | 18,424 | 37,714 | 31,525 |  | SAMN03952824 |
| Tremois_C | 33,991,058 | 3.17 | 29,595,410 | 83.83 | 86.62 | 22,402,376 |  | 18,378 | 38,997 | 35,099 |  | SAMN03952823 |
| Tremois_T | 22,195,046 | 2.07 | 17,963,666 | 76.25 | 85.79 | 13,190,262 |  | 18,821 | 39,065 | 33,210 |  | SAMN03952823 |

Nure_C, Nure in control. Nure_T, Nure in treatment. Tremois_C, Tremois in control. Tremois_T, Tremois in treatment. SNV, single nucleotide variant. *indel*, insertion or deletion variant. The reference genome of *cv*. Morex was obtained from Ensembl Genomes 2013 (http://plants.ensembl.org/info/website/ftp/index.html).
